# Phenotypic Expansion of Autosomal Dominant *LZTR1*-Related Disorders with Special Emphasis on Adult-Onset Features

**DOI:** 10.3390/genes15070916

**Published:** 2024-07-13

**Authors:** Vera Uliana, Enrico Ambrosini, Antonietta Taiani, Sofia Cesarini, Ilenia Rita Cannizzaro, Anna Negrotti, Walter Serra, Gabriele Quintavalle, Lucia Micale, Carmela Fusco, Marco Castori, Davide Martorana, Beatrice Bortesi, Laura Belli, Antonio Percesepe, Francesco Pisani, Valeria Barili

**Affiliations:** 1 Medical Genetics, University Hospital of Parma, 43126 Parma, Italy; 2 Medical Genetics, Department of Medicine and Surgery, University of Parma, 43126 Parma, Italydmartorana@ao.pr.it (D.M.);; 3 Neurology Unit, University Hospital of Parma, 43126 Parma, Italy; 4 Unit of Cardiology, University Hospital of Parma, 43126 Parma, Italy; 5 Regional Reference Centre for Inherited Bleeding Disorders, University Hospital of Parma, 43126 Parma, Italy; 6 Division of Medical Genetics, Fondazione IRCCS-Casa Sollievo della Sofferenza, Viale Cappuccini snc, San Giovanni Rotondo, 71013 Foggia, Italy; 7 Medical Oncology Unit, University Hospital of Parma, 43126 Parma, Italy; 8 Neurosurgery Unit, Head and Neck Department, University Hospital of Parma, 43126 Parma, Italy; 9 Child Neurology and Psychiatry Unit, Department of Human Neuroscience, Sapienza University, Via dei Sabelli 108, 00185 Rome, Italy

**Keywords:** *LZTR1*, schwannomatosis, Noonan syndrome, generalized joint hypermobility

## Abstract

Leucine zipper-like transcription regulator 1 (LZTR1) acts as a negative factor that suppresses RAS function and MAPK signaling; mutations in this protein may dysregulate RAS ubiquitination and lead to impaired degradation of RAS superfamily proteins. Germline *LZTR1* variants are reported in Noonan syndrome, either autosomal dominant or autosomal recessive, and in susceptibility to schwannomatosis. This article explores the genetic and phenotypic diversity of the autosomal dominant *LZTR1*-related disorders, compiling a cohort of previously published patients (51 with the Noonan phenotype and 123 with schwannomatosis) and presenting two additional adult-onset cases: a male with schwannomatosis and Parkinson’s disease and a female with Noonan syndrome, generalized joint hypermobility, and breast cancer. This review confirms that autosomal dominant *LZTR1*-related disorders exhibit an extreme phenotypic variability, ranging from relatively mild manifestations to severe and multi-systemic involvement, and offers updated frequences of each clinical feature. The aim is to precisely define the clinical spectrum of *LZTR1*-related diseases, using also two new emblematic clinical cases. Gaining insight into the mechanisms underneath this variability is crucial to achieve precision diagnostics and the development of therapeutic interventions.

## 1. Introduction

Leucine zipper-like transcription regulator 1 (LZTR1), belonging to the BTB-Kelch proteins, is the substrate-specific adaptor for CUL3 ubiquitin ligase complex [1,2,3].

LZTR1 acts as a negative factor that suppresses RAS function and MAPK signaling; mutations in this protein may deregulate RAS ubiquitination, leading to the impaired degradation of RAS superfamily proteins [2,3,4].

The primary structure of LZTR1 includes six Kelch motifs at the N-terminus and two C-terminal BTB-BACK domains [3]. The Kelch domains selectively recruit substrates, while the BACK domains are considered to mediate dimerization and the binding to CUL3 [4]. Furthermore, the second BTB domain (from N to C) mediates the interaction between the Golgi complex and LZTR1, suggesting that LZTR1 may be a novel Golgi matrix-associated protein [1,4].

*LZTR1* acts as a tumor suppressor gene; for instance, somatic loss-of-function (LOF) variants in *LZTR1* occur in 22% of glioblastomas (high-grade astrocytic neoplasms) [5,6].

It has also been recently identified as a causative gene in RASopathies [7], particularly Noonan syndrome, which can be inherited in an autosomal dominant (Noonan syndrome 10, #MIM 616564) or autosomal recessive (Noonan syndrome 2, #MIM 605275) [8,9,10,11,12,13,14,15,16,17,18] pattern.

Noonan syndrome (NS) is a multisystem condition caused by dysregulation of RAS/MAPK signaling. It is a clinically and genetically heterogeneous entity [14,19]. Clinical features include typical facial dysmorphisms, more pronounced in childhood than in adulthood, skeletal abnormalities, heart defects, and sometimes intellectual disability, coagulation defects, and proliferative disorders. Typical facial features of NS include hypertelorism, downslanting palpebral fissures, epicanthal folds, ptosis, and low-set, posteriorly rotated ears with fleshy helices.

Additionally, *LZTR1* is considered a genetic susceptibility factor for schwannomatosis (#MIM 615670), a rare tumor predisposition syndrome that causes multiple schwannomas [10,20,21,22,23,24,25,26,27,28,29,30,31,32].

Schwannomatosis most likely constitutes a distinct clinical phenotype, as schwannomas are not frequently seen in Noonan syndrome [10,19], although the coexistence of NS and schwannomatosis in patients with *LZTR1* pathogenic variants has been described [7,33]. Moreover, in a reported family with a recessive form, heterozygous carriers showed subtle imaging findings compatible with schwannomas [9]. Interestingly, in an NS patient presenting with schwannomatosis, a *KRAS* mutation has been found [34].

NS-related mutations in *LZTR1* are dominant or loss-of-function variants that occur mainly in the Kelch domains, albeit variants in BTB-BACK domains are also described [1,2,4,35].

Schwannomatosis-associated *LZTR1* variants are mostly LOF changes located in almost every domain [1,20,21].

In this context, we examine the genetic and phenotypic diversity of autosomal dominant *LZTR1*-related disorders by gathering information on a group of previously reported cases. Additionally, we outline adult-onset characteristics in two new patients: a male with schwannomatosis and Parkinson’s disease, and a female with Noonan syndrome, joint hyperlaxity, and breast cancer.

## 2. Materials and Methods

### 2.1. Review

As a preliminary evaluation, the landscape of publicly available databases containing *LZTR1* variants, such as ClinVar, was examined. ClinVar variants with summary evaluations and individual submitter annotations were retrieved from the 10 June 2024 XML file. All alleles associated with the *LZTR1* gene were collected. The variants identified in ClinVar are not all indexed as associated with disease phenotypes; several are related to clinical phenotypic terms which are highly heterogeneous. Thereby, these data were used only for frequency considerations and variant classification, while for phenotype description, we focused on *LZTR1* variants described also in PubMed, which was searched using the MeSH (LZTR1) AND (Noonan Syndrome OR schwannomatosis). A total of 110 publications were retrieved.

Articles written in a different language than English and not-indexed papers were first excluded. Another exclusion criterion was related to the absence of clinical information, especially concerning the clinical diagnosis of schwannomatosis or Noonan Syndrome, or the absence of genetic testing. After exclusions, 25 publications were finally selected. The earliest article was published in February 2014, and the most recent was published in March 2024 (accessed 21 April 2024). Patients with variants that were classified as “benign/likely benign” were removed from the cohort. There were no age or sex limitations.

Variants identified in patients were classified based on the American College of Medical Genetics and Genomics and the Association for Molecular Pathology (ACMG/AMP) guidelines [36] and the Association of Clinical Genomic Science (ACGS) guidelines v4.01 2020.

### 2.2. New Patients Inclusion

In compliance with the local ethical guidelines and the Declaration of Helsinki, the two new patients included in this study provided informed consent for genetic analysis and results publication. Ethical review and approval were waived for this study according to the local policy; informed consent is considered sufficient for reports of an observational nature concerning a limited number of patients.

### 2.3. Neurofibromatosis and Schwannomatosis NGS Panel

The genomic DNA was extracted from peripheral blood using MagCore Genomic DNA Whole Blood Kit, evaluated with Qubit dsDNA High Sensitivity (ThermoFisher, Waltham, MA, USA).

The analysis is based on the records of the Unit of Medical Genetics of the Parma University Hospital, reporting the results of the genetic testing performed by next-generation sequencing using the Illumina MiSeq platform (TruSeq Custom Amplicon v.1.5), with a gene panel including *NF1* (GenBank reference sequence, RefSeq: NM_001042492), *SPRED1* (RefSeq: NM_152594), *NF2* (RefSeq: NM_016418.5), *LZTR1* (RefSeq: NM_006767.4), and *SMARCB1* (RefSeq: NM_003073.5) on patients with suspect of neurofibromatosis spectrum.

### 2.4. Noonan Syndrome Analysis

Genomic DNA analyzed at the Division of Medical Genetics, Fondazione IRCCS-Casa Sollievo della Sofferenza, was extracted from peripheral blood by using Bio Robot EZ1 (Qiagen, Hilden, Germany), according to standard procedures. The DNA was quantified with a Nanodrop 2000 C spectrophotometer (ThermoFisher Scientific, Waltham, MA, USA). DNA underwent next-generation sequencing (NGS) with a custom-made SureSelect gene panel (Agilent Technologies, Santa Clara, CA, USA) designed to selectively capture known genes associated with a wide range of Noonan syndromes and their major differential diagnoses including: *BRAF* (RefSeq: NM_004333), CBL (RefSeq: NM_005188), *CDC42* (RefSeq: NM_001039802), *HRAS* (RefSeq: NM_005343.4), *KRAS* (RefSeq: NM_033360), *LZTR1* (RefSeq: NM_006767.4), *MAP4K4* (RefSeq: NM_145686), *MAPK1* (RefSeq: NM_002745), *MEK1/MAP2K1* (RefSeq: NM_002755), *MEK2/MAP2K2* (RefSeq: NM_030662), *MRAS* (RefSeq: NM_012219.4), *NF1* (RefSeq: NM_001042492), *NRAS* (RefSeq: NM_002524), *PPP1CB* (RefSeq: NM_002709.3), *PTPN11* (RefSeq: NM_002834), *PTPN12* (RefSeq: NM_002835), *RAF1* (RefSeq: NM_002880), *RIT1* (RefSeq: NM_006912.5), *RRAS* (RefSeq: NM_006270), *RRAS2* (RefSeq: NM_012250.6), *SHOC2* (RefSeq: NM_007373), *SOS1* (RefSeq: NM_005633), *SOS2* (RefSeq: NM_006939.4), *SPRED1* (RefSeq: NM_152594), and *SPRED2* (RefSeq: NM_181784). Libraries were prepared using the SureSelect target enrichment kit (Agilent Technologies, Santa Clara, CA, USA) following manufacturer’s instructions. Targeted fragments were then sequenced on NextSeq 500 platform (Illumina, San Diego, CA, USA) using a NextSeq 500 mid output kit V2.5 (300 cycles flow cell). Sequences were automatically demultiplexed and results were written to FASTQ files. Reads were quality-checked, trimmed, mapped to the GRCh37 (hg19) reference assembly with Burrows-Wheeler Alignment tool (BWA), and deduplicated, and variants called by Alissa Align & Call (Agilent Technologies, Santa Clara, CA, USA). Candidate variants were confirmed by Sanger sequencing. In case of available relatives, segregation was carried out by Sanger sequencing.

## 3. Results

### 3.1. Review: LZTR1 in Noonan Syndrome 10

**Figure 1:**
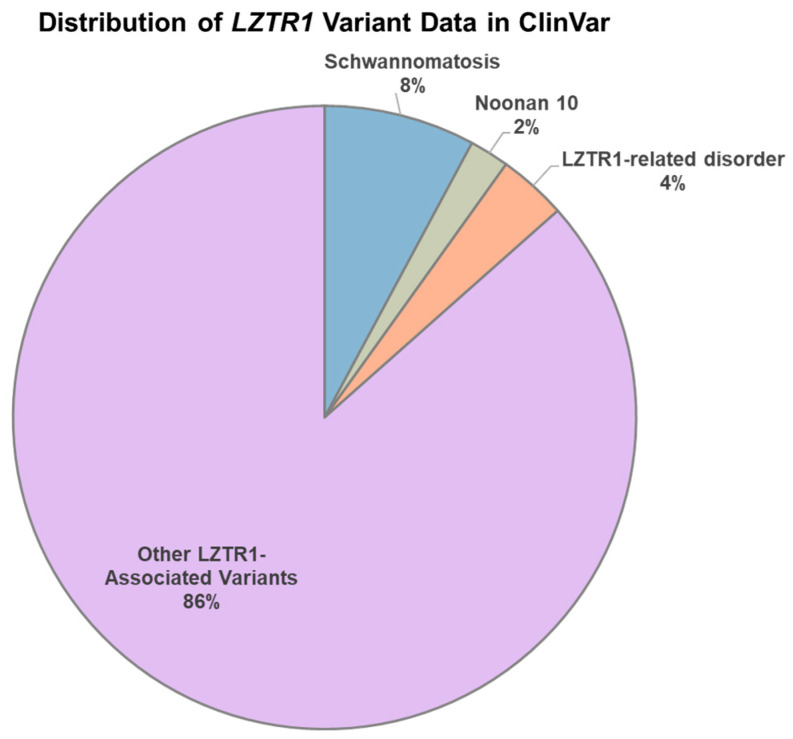
Pie chart exhibiting *LZTR1* variants indexed on ClinVar database as Noonan syndrome 10, schwannomatosis, and *LZTR1*-related disorders, majority of which are not indexed as associated with disease phenotypes.

A total of 3263 *LZTR1* variants were inspected in the ClinVar database. By investigating the pathogenicity score, we found that 682 variants are pathogenic, 260 changes are likely pathogenic and 1686 are indexed as variants of uncertain significance. By filtering out all of the variants which are not indexed as associated with disease phenotypes, we identified 116 alterations indexed as *LZTR1*-related disorders. There are 67 variants associated specifically with the term “Noonan syndrome 10” (Figure 1).

Next, we focused on those variants which are described in the literature on PubMed, to obtain the proper clinical phenotypic characterization. We identified a total 51 patients with variants in *LZTR1* and a clinical diagnosis of Noonan syndrome; of these, 28 are female and 20 are male, while for 3 patients, the sex was not reported. The median age is 17.2 years, the youngest patient is 2 years old, and the oldest is 69 years old. In these 51 patients, a total of 30 variants have been identified, since some of the patients are related and/or harbor the same variant.

Most of the cases exhibited short stature (68.3%), in accordance with the Noonan phenotype, skeletal abnormalities (58%), and cardiac defects (66%) (Table 1). Interestingly, 32% of the cases featured global developmental delay and 27% featured abnormal hemostasis (Table 1). Conversely, a few cases displayed neoplasms (n = 4), schwannomas (n = 1), cafe-au-lait spots (n = 6), and lymphedema (n = 2) (Table 1). Comparing the clinical characteristics reported in the literature for classic Noonan Syndrome (NS) patients to those of the patients affected by *LZTR1*-related Noonan Syndrome 10, a similar phenotype is evident (Supplementary Table S1). Some features, such as hearing loss, renal anomalies, learning disabilities, and joint hyperextensibility, appear to be more common in NS and are generally not taken into consideration in patients with *LZTR1* variants [37].

**Figure 2:**
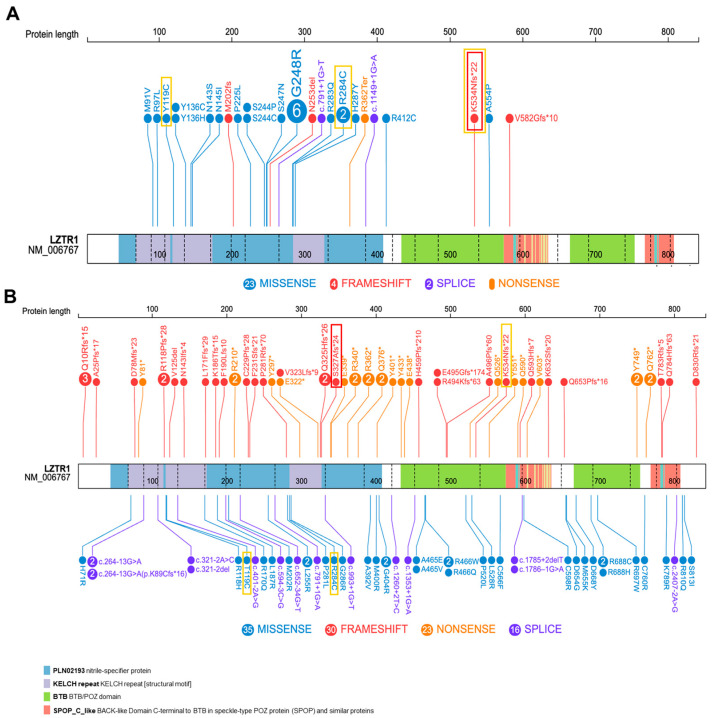
LZTR1 protein representation displaying variants described in literature-based cases affected by Noonan syndromes (**A**) and by schwannomatosis (**B**). Numbers within circles correspond to variants mentioned in Table 1 and Table 2. Red boxes indicate our cases. Yellow boxes specify common variants in two patients’ cohorts. Circle colors are indicative of variant type (missense, frameshift, nonsense, and splicing), while protein-domain regions are depicted using distinct colors.

By investigating the molecular alteration type, missense variants (23 out of 30) represent the largest category (Figure 2A) in this cohort of patients with the Noonan phenotype, while the remaining are frameshift (n = 4), nonsense (n = 1), and splice-site-related variants (n = 2). As expected, these variants lie within the protein domains with the Kelch repeats (Figure 2A). More than half of the variants in the Noonan cohort are classified as pathogenic alterations (n = 18/30). Together with those belonging to class 4 (9 out of 30), they represent the majority of the changes, while variants of uncertain significance are in a limited number (3 out of 30).

### 3.2. Review: LZTR1-Related Schwannomatosis

The *LZTR1*-related schwannomatosis variants indexed in the ClinVar database comprise 256 out of a total of 3263 *LZTR1* alterations (Figure 1). To better investigate the affected patients, we focused on published cases on Pubmed which comprise 123 eligible schwannomatosis patients with a total of 105 germline *LZTR1* variants (Table 2). This patient cohort includes 34 males and 37 females (the remaining ones were not specified) with ages spanning from 15 to 70 years and a median age of 40 years.

By inspecting the type of molecular changes, a comparable number of missense (35 out of 105), frameshift (30 out of 105), and nonsense (23 out of 105) variants were observed in patients with schwannomatosis (Figure 2B). 15% of the schwannomatosis cases exhibited variants affecting splice-site regions (16 out of 105) (Figure 2B and Table 2). Interestingly, only one reported case in the cohort of patients with schwannomatosis was characterized by a genomic deletion of the entire LZTR1 gene, c.1-?2523+?del (Table 2).

Patients with *LZTR1* schwannomatosis exhibited 44 pathogenic variants, 51 likely pathogenic alterations, and 10 VUS (Table 2) which, as described, may be associated with loss-of-function mutations predisposing to schwannomatosis.

Only three variants (Figure 2 in yellow boxes) are reported in both Noonan syndrome and schwannomatosis patients (Table 1 and Table 2), which confirm clinical heterogenous phenotypes related to *LZTR1* variants.

Familiar cases are described with incomplete penetrance and variable expressivity, with an inheritance which most frequently occurs in an autosomal dominant manner.

### 3.3. New Case #1

Patient 1, a 55-year-old male, was first admitted to our Medical Genetics Unit on suspicion of neurofibromatosis.

**Figure 3:**
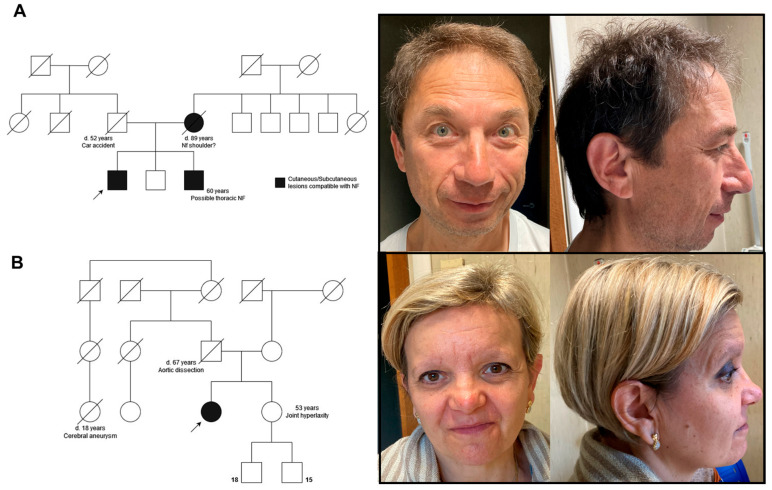
The families and facial features of the patients. On the left, family pedigrees of case 1 (**A**) and case 2 (**B**). On the right, front and lateral views of the probands described as case 1 (**A**) and case 2 (**B**). Arrows indicate the probands.

His family history revealed a café-au-lait spot (CALS) on his deceased mother, two CALSs on his brother, and unspecified tremors in both his maternal grandfather and a maternal uncle (Figure 3A).

At 47 years old, he was admitted to the department of neurology for tremor in his right hand that had appeared a year earlier. Neurological examination showed a bilateral resting and postural tremor of the upper limbs and mild motor impairment, predominantly on the right side leading to a diagnosis of Parkinson’s disease. The brain MRI was normal. Treatment with a dopamine agonist was initiated, resulting in the initial improvement of motor symptoms. However, l-dopa was added in subsequent years due to a gradual loss of benefit. An EMG showed neurogenic pluriradicular impairment without signs of denervation in L5 and S1.

At the age of 55, he was admitted to the neurosurgery department because of lumbar pain. An MRI of the vertebral column identified at least 12 spinal formations referred to as neurofibromas; an L3 intradural neurofibroma was histologically diagnosed as a schwannoma; discal protrusion and mild spondylolystesis were also reported. A schwannoma of the left arm was diagnosed at 55 years old. The ophthalmologic examination was normal.

At our clinical examination, he presented with macrocephaly (OFC 59.5 cm, >97th percentile) and normal height (176 cm, 50–75th percentile) and weight (75 Kg, BMI 24). He had two cafe-au-lait spots larger than 1.5 cm in diameter (one on the back and one on the ankle), several subcutaneous neurofibromas (about five on the scalp, one on the left arm, and two on the left foot), and no axillary or inguinal freckling.

The NGS analysis using a panel including genes associated with schwannomatosis identified the heterozygous variant NM_006767: c.979delA (p.Ser327Alafs*24) in the *LZTR1* gene. The variant was rated as likely pathogenic according to the American College of Medical Genetics and Genomics (ACMG) criteria by attributing PVS1 (a null variant in a gene whose loss of function is a known mechanism of disease) and PM2 (absent from control). It is not reported in ClinVar or in the literature as associated with *LZTR1*-related disorders. However, a frameshift variant involving the adjacent amino acid residue (c.975delG; p.Gln325Hisfs*26) has been found in patients with schwannomatosis [38,40,41]. Unfortunately, no relatives were available for testing.

The patient also underwent an NGS analysis focused on Parkinson’s disease (including the following genes: ATP13A2, DNAJC13, DNAJC6, EIF4G1, FBX07, GBA, GCH1, GYGYF2, GRN, HTRA2, LRRK2, MAPT, PARK2, PARK7, PINK1, PLA2G6, SNCA, VPS35, SYNJ1, UCHL1, TMEM230, RAB29B); neither pathogenetic/probably pathogenetic nor unknown significance variants were identified.

### 3.4. New Case #2

Patient 2, female, was 51 years old when she was referred to our Medical Genetics Unit for a possible heritable disorder of connective tissue.

Her family history revealed that her mother had breast cancer at 70 years of age and a vertebral schwannoma at S1–S2, her father died of aortic dissection at the age of 67, and a paternal cousin died from a suspected cerebral hemorrhage at 18 years old. She has a sister with joint hyperlaxity but no other pathological signs or symptoms (Figure 3B).

Normal psychomotor development with dyslexia was reported in childhood.

Given a history of easy bruising since childhood and menorrhagia, she accessed the hemophilia center of Parma hospital and was diagnosed with a congenital platelet function disorder at 40 years old.

She also presented with hypothyroidism due to Hashimoto’s thyroiditis, endometriosis since the age of 31 years, and degenerative retinopathy.

At 47 years old, she was referred to the physiatrist for left knee dislocation and chronic joint pain; bilateral flat feet treated with arch supports, hallux valgus, weak knees, and joint laxity were also reported.

At 51 years old, a cardiologic examination was performed; she did not report chest pain or dyspnea at rest and no anomalies were noted in the ECG. The echocardiographic exam showed myxomatous mitral valve leaflets and a diverticulum of the basal inferior interventricular septum. A heart CT scan and MRI confirmed the presence of ventricular crypts without shunts.

At 53 years old, an invasive ductal carcinoma of the left breast was diagnosed (G3, stage IA T1N0M0, Er+, 50%, Pr+, 30%, HER2 3+), and it was treated with neoadjuvant and adjuvant therapy with Trastuzumab and Pertuzumab, quadrantectomy, and radiotherapy. She underwent adjuvant aromatase inhibitor therapy; joint pain and osteoporosis were reported as side effects.

At our clinical examination, she was 160 cm tall (25–50th percentile), had relative macrocephaly (head circumference 55 cm, 75–90th percentile) and weighed 66 kg (BMI 25.8). Hypertelorism, mild pterigium, two cafe-au-lait spots larger than 1.5 cm (one on the left forearm and one on the right calf), kyphoscoliosis, mild skin hyperextensibility, and bilateral ptosis were detected. The patient scored 7 on the Beighton scale (scores ≥ 5 indicate hypermobility). No atrophic scars, arachnodactyly, or skin streaks were noted.

The NGS analysis using a panel including genes associated to RASopathies identified the heterozygous variant NM_006767: c.1602del (p.Lys534Asnfs*22) in *LZTR1*. The variant was rated as pathogenic according to the American College of Medical Genetics and Genomics (ACMG) criteria by attributing PVS1 (a null variant in a gene whose loss of function is a known mechanism of disease), PM2 (absent from control), and PP5 (a variant recently reported by a reputable source as pathogenic). In the ClinVar database, this variant is classified as pathogenic (two stars), and it has been cited in the literature as being associated with *LZTR1*-related disorders [19,27]. Its frequency in population databases is extremely low (allelic frequency 1:152204 in gnomAD). It has been reported in patients with schwannomatosis, but also in healthy relatives [23,42].

By investigating within the reviewed Noonan patients’ cohort (Table 1), the frameshift variants are few, 4 out of 30, compared to other change types, and they are interspersed throughout the entire gene (Figure 2A). No relatives were available for testing.

Considering the clinical features (including facial features, the heart defect, the bleeding tendency, and the platelet disorder already reported in association with RASopathies [28,29]) and the result of genetic testing, a diagnosis of autosomal dominant Noonan syndrome was made.

The NGS analysis using a panel including genes associated with hereditary breast and ovarian cancers (SOPHiA HEREDITARY CANCER SOLUTION™ kit, including the following genes: *ABRAXAS1*, *ATM*, *APC*, *BARD1*, *BRCA1*, *BRCA2*, *BRIP1*, *CDH1*, *CHEK2*, *EPCAM*, *MLH1*, *MRE11*, *MSH2*, *MSH6*, *MUTYH*, *NBN*, *PALB2*, *PIK3CA*, *PMS2*, *PMS2CL(1)*, *PTEN*, *RAD50*, *RAD51C*, *RAD51D*, *STK11*, *TP53*, and *XRCC2*) resulted non-informative; neither pathogenic/probably pathogenic nor unknown significance variants were identified.

## 4. Discussion

*LZTR1*-related phenotypes are not yet systematically classified and characterized, mainly due to their heterogeneity. Germline *LZTR1* variants are reported in Noonan syndrome, which can be either autosomal dominant (Noonan syndrome 10, #MIM 616564) or autosomal recessive (Noonan syndrome 2, #MIM 605275), and are also associated with susceptibility to schwannomatosis (#MIM 615670).

The autosomal dominant Noonan syndrome associated with the *LZTR1* gene displays incomplete penetrance and variable expressivity [35]. Commonly described features in Noonan syndrome 10 include typical facial features, short stature, relative macrocephaly, and, in some patients, heart defects (such as hypertrophic cardiomyopathy, pulmonary stenosis, and others), coagulation deficits, ectodermal anomalies, and intellectual disability [35].

Schwannomatosis is part of the neurofibromatosis spectrum, along with *NF1*- and *NF2*-related conditions. The phenotypic overlap between neurofibromatosis type 1 and Noonan syndrome is well known; some patients with pathogenetic variants in NF1 gene exhibit a Noonan syndrome phenotype [28,29], likely due to the interaction between neurofibromin and the RAS pathway [43,44]. The *LZTR1* gene, which also interacts with the RAS pathway, is associated with both NS and schwannomatosis [2,3,4]. Patients with these two different phenotypes may harbor different types of variants (dominant negative versus loss-of-function) localized in different domains [1,2,4,20,21,35]. A detailed examination of *LZTR1* variants associated with autosomal dominant Noonan syndrome (NS) suggests that this gene is functionally linked to the RAS/MAPK pathway by negatively regulating RAS protein levels and MAPK signaling, typically acting as dominant negative mutations. These dominant negative variants interfere with the normal function of LZTR1, leading to an increase in RAS protein levels and hyperactivation of the MAPK signaling pathway. This hyperactivation contributes to the clinical manifestations of Noonan syndrome by dysregulating cellular growth and development. In contrast, loss-of-function variants of *LZTR1*, which are commonly associated with schwannomatosis, result in the complete inactivation of the gene. This loss of function leads to a lack of negative regulation of the RAS and MAPK signaling pathways, causing aberrant cell proliferation and the development of benign schwannomas.

The different impacts of *LZTR1* variants highlight the gene’s crucial role in modulating RAS signaling. Dominant negative and hyperactive mutations lead to excessive pathway activation, causing developmental disorders like Noonan syndrome, whereas loss-of-function mutations lead to tumorigenesis, such as schwannomatosis.

Moreover, *LZTR1* has been recently reported in association with congenital malformations, particularly bladder exstrophy and mitral valve prolapse, as well as cancers, including breast cancer, ependymoma, and leukemia [30,31,32,33,34,35]. Even though clear evidence of these connections is still lacking, it is interesting to note the wide range of clinical features reported in patients with *LZTR1* variants.

We reported two patients with autosomal dominant *LZTR1*-related conditions with unique features not previously reported.

The first is a male patient with a frameshift variant in the Kelch domains, presenting a phenotype compatible with schwannomatosis, and early-onset Parkinson’s disease, without any other pathogenic variants explaining the neurological phenotype.

The LZTR1 gene plays a role in the central nervous system [1]. LZTR1 interacts with CUL3 and neurofibromin 1 (NF1) to regulate nighttime sleep by increasing GABA receptor signaling and has been associated with RAS-related neurological diseases caused by NF1 deficiency [1,45]. NF1 is a negative regulatory factor of the RAS/MAPK signaling pathway, which, together with LZTR1, may inhibit RAS/MAPK signaling pathways [1,46].

Neurodevelopmental delay is a known feature of *LZTR1*-related diseases, affecting almost 32% of the patients reported in the literature, as shown in our review.

Interestingly, it has been reported that the functions of LZTR1 in the nervous system are not restricted to those described above and may be closely related to neurodegenerative diseases, such as Alzheimer’s disease and Parkinson’s disease [1,46].

The initial report of Parkinson’s disease in our case could stem from the fact that *LZTR1*-related disorders are primarily reported in pediatric patients, and Parkinson’s disease is potentially a late-onset feature. We consider two possible explanations for this co-occurrence: our patients might present with *LZTR1*-related conditions and independently have Parkinson’s disease; alternatively, *LZTR1* pathogenic variants could confer susceptibility to Parkinson’s disease.

The second case is a female patient with a heterozygous pathogenic frameshift *LZTR1* variant in the BTB-BACK domains and a phenotype compatible with Noonan syndrome, associated with generalized joint hypermobility and breast cancer.

Generalized joint hypermobility is more likely to be associated with a genetic syndrome compared to localized joint hypermobility [47,48]. In Noonan syndrome, joint hypermobility is reported mostly in studies considering pain in adult patients, and when tested, is found in about 50% of the patients [49,50]. Our patient was initially tested for joint hypermobility due to the suspicion of a connective tissue disorder. Ultimately, including *LZTR1* testing in patients with hypermobility could help clarify this possible correlation.

Concerning cancer risk, RASopathies are actually cancer-prone disorders [51]. The cumulative risk of cancer in NS is evaluated at 4% by age 20 [52,53,54], although breast cancer is rarely reported in NS [52]. *LZTR1* variants have been associated with proliferative disorders, such as acute lymphoblastic leukemia in two NS patients [9], oligo-astrocytoma in one NS patient [6], and glioblastoma in two schwannomatosis patients [39].

The second patient had breast cancer at 53 years of age, but the lifetime risk of developing breast cancer is high, occurring in about 1 in 8 women in some Western countries [55], so we can suppose a fortuitous association. However, due to the higher risk of cancer in NS, the reported *LZTR1* somatic mutations in breast cancer (COSMIC database, http://www.sanger.ac.uk/cosmic, accessed on 8 July 2024), and the reported association between *LZTR1* and breast cancer in a recent study [56,57], we cannot completely exclude a *LZTR1*-related increase in cancer risk.

For both of the *LZTR1*-related phenotypes, we observed a comparable number of female and male patients. No statically significant correlation has been found between assigned sex at birth and clinical manifestations. Considering available data regarding age and sex, female patients with *LZTR1* schwannomatosis are referred at a slightly younger age (average 37.2 years) compared to males (42 years), but the difference is not significant (*p* = 0.07) and the data may not be reliable since a schwannoma is usually present years before the official diagnosis. In both sexes, there are reports of lesions identified during adolescence, the youngest being at 15 years of age.

Even establishing correlation between genotype and phenotype is challenging. As previously reported, variants associated with autosomal dominant Noonan syndrome are almost all missense variants (77%), with the Kelch domain being mainly affected (87% of the variants). Examining possible hotspots, the same missense variant (p.Gly248Arg) is reported in six families with autosomal dominant NS. Five other NS patients from three different families harbor missense variants in adjacent positions (p.Ser247Asn, p.Ser244Cys, p.Ser244Pro). Conversely, it is harder to define a clear hotspot in relation to schwannomatosis; variants are reported in all known domains of the protein and include all type of variants, with truncating variants making up 50%.

Both of our patients present frameshift variants, which are more frequently described in schwannomatosis than in Noonan syndrome (Figure 2A,B). The variant identified in the second patient with Noonan syndrome is located outside the Kelch domain and has been previously reported in cases of schwannomatosis; see Table 2 [23,42]. Interestingly, the untested mother of our Noonan patient had a schwannoma in the S1–S2 area and did not seem to present any NS-specific phenotype, further highlighting the heterogeneity of clinical phenotypes.

Other cases of recurrent heterozygous variants associated with both NS and schwannomatosis have been reported in the literature, such as p.R284C [7,23], K534fs, and G248R [35] (Figure 2A,B). Other genetic or non-genetic unknown factors could contribute to the different phenotypes, even within the same family. This clinical heterogeneity complicates the process of establishing clear genotype–phenotype correlations.

Finally, another complicated issue is the interpretation of variants. More than half of the 3263 variants reported in ClinVar are categorized as VUS. Besides the lack of functional data, this huge uncertainty could also be due to the difficulty of attributing the unspecific phenotype of a patient to a single *LZTR1* variant. In fact, the dual mode of transmission may raise doubts, since a variant could be reported as (likely) pathogenic, but only in a homozygous or compound heterozygous state (PM3 criteria from ACMG). One could even argue that, in some NS patients with a heterozygous *LZTR1* variant, a second variant was missed due to technical limits (e.g., deep intronic variants).

To try to solve these issues, a joint effort of clinical and laboratory genetics is needed. On the clinical side, family history and segregation are important to determine the inheritance and the applicability of certain ACMG criteria, such as “de novo data” (PS2 or PM6) and “allelic data” (PM3). Increasing the number of patients reported with a detailed description could help refine the phenotype. On the laboratory side, more comprehensive studies like Whole Genome Sequencing and Long-Read Sequencing can cover the technical limits mentioned above (e.g., missing “in trans” variants), while analyzing the affected tissues, like schwannomas, could identify a somatic second hit or potential driver mutations in other related genes.

In conclusion, there are still many open issues concerning *LZTR1*-related diseases. First, it is unclear whether Noonan syndrome and schwannomatosis are two distinct manifestations of *LZTR1* disease or part of the same continuous spectrum. Moreover, we expect a phenotype expansion as new diagnoses are made in adult patients, revealing novel features not commonly seen in pediatric patients. Parkinson’s disease and breast cancer could constitute possible associations, even if a phenotype expansion cannot be drawn from single case reports. More molecular evidence is surely needed, and the descriptions of larger cohorts of adult patients harboring *LZTR1* pathogenic variants, including parents and family members of known pediatric patients, could help clarify the real extent of the *LZTR1*-related phenotype [58].

## Figures and Tables

**Table 1:** Clinical and genetic findings in autosomal dominant Noonan syndrome associated with *LZTR1* variants reported in literature. Fifty-one patients exhibited 30 constitutional *LZTR1* variants in blood and showed a heterogenous clinical phenotype. Majority of variants are pathogenic (P) and likely pathogenic (LP) according to ACMG criteria. Our case is highlighted in grey. Yellow boxes specify common variants in two patients’ cohorts.

Ref.	Variants (n = 30)	Classification (P = 18/LP = 9/VUS = 3)	Patients (n = 51)	Gender (28F/20M)	Age	Noonan Syndrome (n = 48/51)	Schwannomas (n = 1/48)	Short Stature (n = 27/42)	Relative Macrocephaly (n = 7/14)	Noonan Facial Features (n = 45/45)	Skeletal Anomalies (n = 25/43)	Cardiac Defects (n = 27/41)	Developmental Delay (n = 14/44)	Abnormal Hemostasis (n = 9/33)	Lymphedema (n = 2/34)	Cancer (n = 4/45)	Cafe-au-Lait Spots (n = 6/42)	Lentigines/Nevus (n = 3/42)	Ectodermal Findings (n = 19/43)	Other (n=17)
Yamamoto et al., 2015 [7]	c.742G>A, p.Gly248Arg	P	Proband	F	11	+	-	+	n.a.	+	+	HCM	-	-	-	-	-	-	-	lacrimal duct obstruction
			Mother	F	45	+	-	+	n.a.	+	+	MVP	-	-	-	-	-	-	-	
			Grandfather	M	69	+	-	+	n.a.	+	+	MVP	-	+	-	-	-	-	-	
	c.850C>T, p.Arg284Cys	P	Proband	F	14	+	-	+	n.a.	+	-	-	-	-	-	-	-	-	-	
			Mother	F	38	+	-	+	n.a.	+	-	-	-	-	-	-	-	-	+	
			SIbling1	M	15	+	-	+	n.a.	+	-	-	-	-	-	-	-	+	+	
			Sibling2	F	12	+	-	-	n.a.	+	-	n.a.	-	-	-	-	-	-	+	hemangioma
			Half sibling1	F	16	+	-	+	n.a.	+	-	-	-	-	-	-	-	-	-	
			Haf sibling2	F	3	+	-	+	n.a.	+	-	-	-	n.a.	-	-	-	-	-	
	c.859C>T, p.His287Tyr	LP	Proband	M	16	+	-	-	n.a.	+	-	PS	+	+	-	-	-	-	+	Hyperopia; cryptorchidism
	c.356A>G, p.Tyr119Cys	LP	Proband	F	30	+	-	-	n.a.	+	-	HCM	-	-	+	-	-	-	-	
	c.740G>A, p.Ser247Asn	LP	Proband	M	18	+	-	-	n.a.	+	+	MVP	+	-	-	-	-	-	+	
			Mother	F	53	+	+	+	n.a.	+	-	other	-	-	-	2 neurinomas, 1 schwannoma	-	-	-	hyperopia
Jacquinet et al., 2020 [6]	c.850C>T, p.Arg284Cys	P	Proband	M	26	+	-	+	+	+	+	-	+	+	-	glioblastoma	-	-	-	cryptorchidism
			Mother	F	n.a.	+	-	+	-	+	-	n.a.	-	-	-	-	-	-	-	
Zhao et al., 2021 [11]	c.1149+1G>A, p. (?)	P	Proband	F	6	+	-	+	n.a.	+	+	-	+	n.a.	n.a.	-	+	-	+	
			Mother	F	27	+	-	+	n.a.	+	-	-	-	n.a.	n.a.	-	-	-	-	
			Sister	F	2	+	-	+	n.a.	+	+	-	-	n.a.	n.a.	-	-	-	-	
Pagnamenta et al., 2019 [14]	c.406T>C, p.Tyr136His	P	Proband	F	8	+	-	+	n.a.	+	-	other	-	n.a.	n.a.	-	-	-	-	hepatomegaly, Malrotation of small bowel
	c.434A>T, p.Asn145Ile	LP	Proband	M	20	+	-	-	n.a.	+	+	PS	+	+	-	-	+	-	-	
			Mother	F	n.a.	+	-	n.a.	n.a.	+	-	n.a.	+	+	-	-	-	-	-	
			Uncle	F	n.a.	+	-	-	n.a.	+	-	n.a.	-	-	-	-	-	-	-	delayed puberty, hypothyroidism
			Cousin1	M	n.a.	+	-	n.a.	n.a.	+	-	-	+	-	-	-	-	-	-	periventricular leukomalacia,
			Cousin2	F	n.a.	+	-	-	n.a.	+	+	MVP	-	-	-	-	-	-	-	
	c.290G>T, p.Arg97Leu	P	Proband	F	14	+	-	+	n.a.	+	+	HCM/VSD	+	+	-	-	-	-	+	
	c.407A>G, p.Tyr136Cys	P	Proband	M	5	+	-	+	n.a.	+	+	PS	+	-	-	-	-	-	+	cryptorchidism
	c.731C>G, p.Ser244Cys	P	Proband	M	7	+	-	+	n.a.	+	+	-	-	-	-	-	+	-	-	single transverse palmar crease
	c.742G>A, p.Gly248Arg	P	Proband	F	3	+	-	+	n.a.	+	+	other	+	-	-	-	-	-	+	perinatal asphyxia,
Umeki et al., 2019 [35]	c.848G>A, p.Arg283Gln	LP	Proband	M	11	+	-	+	-	+	+	ASD/VSD	+	-	-	-	+	-	+	
	c.742G>A, p.Gly248Arg	P	Proband	M	2	+	-	-	-	+	-	other	-	n.a.	n.a.	-	-	-	-	
			Mother	F	n.a.	+	-	n.a.	n.a.	+	n.a.	VSD	-	n.a.	n.a.	-	n.a.	n.a.	n.a.	
	c.428A>G, p.Asn143Ser	VUS	Proband	M	8	+	-	+	+	+	+	HCM	+	-	-	-	-	+	+	cryptorchidism
			Father	M	n.a.	-	-	n.a.	n.a.	n.a.	n.a.	n.a.	n.a.	n.a.	n.a.	n.a.	n.a.	n.a.	n.a.	
c.604_605del, p.Met202fs	c.606_650del, p.Met202fs	P	Proband	F	5	+	-	n.a.	+	+	+	HCM/PS	-	-	-	-	-	-	+	
			Father	M	n.a.	-	-	n.a.	n.a.	n.a.	n.a.	n.a.	n.a.	n.a.	n.a.	n.a.	n.a.	n.a.	n.a.	
	c.756_758del, p.Asn253del	VUS	Proband	M	11	+	-	+	+	+	+	HCM	+	-	-	-	-	-	+	optic atrophy
	c.1660G>C, p.Ala554Pro	VUS	Proband	F	16	+	-	-	+	+	+	HCM	+	n.a.	n.a.	-	-	+	+	
			Father	M	n.a.	-	-	n.a.	n.a.	n.a.	n.a.	n.a.	n.a.	n.a.	n.a.	n.a.	n.a.	n.a.	n.a.	
Guemes et al., 2019 [13]	c.742G>A, p.Gly248Arg	P	Proband	M	5	+	-	+	-	+	+	PS	-	-	-	-	-	-	+	choroid plexus cyst; cryptorchidism
	c.730T>C, p.Ser244Pro	LP	Proband	M	8	+	-	+	n.a.	+	+	-	-	+	-	-	-	-	+	chiari malformation type 1
			Mother	F	n.a.	+	-	+	n.a.	+	+	-	-	n.a.	n.a.	-	n.a.	n.a.	+	
Carcavilla et al., 2023 [15]	c.742G>A, p.Gly248Arg	P	Proband	n.a.		+	n.a.	n.a.	n.a.	n.a.	n.a.	n.a.	n.a.	n.a.	n.a.	n.a.	n.a.	n.a.	n.a.	
	c.791+1G>T, p.(?)	P	Proband	n.a.		+	n.a.	n.a.	n.a.	n.a.	n.a.	n.a.	n.a.	n.a.	n.a.	n.a.	n.a.	n.a.	n.a.	
	c.1084 C>T, p.Arg362Ter	P	Proband	n.a.		+	n.a.	n.a.	n.a.	n.a.	n.a.	n.a.	n.a.	n.a.	n.a.	n.a.	n.a.	n.a.	n.a.	
Unuma et al., 2023 [16]	c.1234C>T, p.Arg412Cys	LP	Proband	M	5	+	-	-	n.a.	+	n.a.	Other	n.a.	n.a.	n.a.	Leukemia	n.a.	n.a.	n.a.	
Chaves Rabelo et al., 2022 [8]	c.742G>A, p.Gly248Arg	P	Proband	F	10	+	-	+	-	+	+	PS	-	-	-	-	+	-	-	
	c.674C>T, p.Pro225Leuc	LP	Proband	F	13	+	-	-	-	+	+	PS/MVP	-	+	+	-	-	-	-	cystic hygroma
			Mother	F	41	+	-	-	-	+	+	-	-	-	-	-	-	-	-	
Kraoua et al., 2022 [17]	c.1745delT, p.Val582Glyfs*10	P	Proband	M	4	+	-	+	+	+	+	HCM	-	n.a.	n.a.	-	-	-	+	
Alkaya et al., 2021 [18]	c.271A>G, p.Met91Val	LP	Proband	F	6	+	-	-	n.a.	+	-	PS	-	n.a.	n.a.	-	-	-	-	
Current Case 2	c.1602del, p.Lys534Asnfs*22	P	Proband	F	51	+	-	-	+	+	-	MVP	-	+	-	Breast cancer	+	-	+	generalized joint hypermobility

Legends: HCM: Hypertrophic cardiomyopathy, PS: Pulmonary stenosis, MVP: Mitral Valve prolapse, AC: aorta coartation, complex malformation, VSD: ventricular spectal defect, ASD: atrial septal defect, n.a.: not available, +: presence of the clinical feature, -: absence of the clinical characteristic.

**Table 2:** Total schwannomatosis probands associated with *LZTR1* variants reported in literature. Total of 123 patients affected with schwannomas showed 105 constitutional *LZTR1* variants in blood with a pathogenicity class belonging to class 4 and 5 according to ACMG criteria. Our case is highlighted in grey. Yellow boxes specify common variants in two patients’ cohorts. n.a.: not available, +: presence of the clinical feature.

Ref.	Variants (n = 105)	Classification (P/LP/VUS)	# Patients (n = 123)	Gender (M = 34/F = 37)	Age	# Pt with Schwannomas
Piotrowski et al. (2014) [20]	c.264-13G>A, p.Lys89Cysfs*16	LP	Proband	M	n.a.	+
			Son	M	n.a.	+
			Daughter	F	n.a.	+
	c.356A>G, p.Tyr119Cys	LP	Proband	M	n.a.	+
			Father	M	n.a.	+
	c.1559C>T, p.Pro520Leu	LP	Proband	M	n.a.	+
			Mother	F	n.a.	+
	c.2062C>T, p.Arg688Cys	LP	Proband	F	n.a.	+
	c.2438G>T p.Ser813Ile	LP	Proband	F	n.a.	+
	c.27delG, p.Gln10Argfs*15	P	Proband	F	n.a.	+
	c.2348_2351del, p.Thr783Argfs*5	P	Proband	F	n.a.	+
			Father	M	n.a.	+
			Brother	M	n.a.	+
	c.594-3C>G, p.(?)	LP	Proband	F	n.a.	+
	c.1397G>A, p.Arg466Gln	P	Proband	M	n.a.	+
			Daughter	F	n.a.	+
Hutter et al. (2014) [24]	c.321-2delA, p.(?)	P	Proband	F	19	+
	c.352dupC, p.Arg118Profs*28	P	Proband	F	51	+
	c.1312G>T, p.Glu438*	P	Proband	F	56	+
	c.1480_1481insAG, p.Arg494Lysfs*63	LP	Proband	F	40	+
	c.2247C>A, p.Tyr749*	P	Proband	F	46	+
Paganini et al. (2015) [23]	c.212A>G, p.His71Arg		Proband	F	34	+
	c.243T>G, p.Tyr81*	LP	Proband	M	32	+
	c.352dupC, p.Arg118Profs*28	P	Proband	F	49	+
	c.373_375del, p.Val125del	LP	Proband	F	46	+
	c.513delG, p.Leu171Phefs*29	LP	Proband	F	34	+
	c.555_556dupCA, p. Lys186Thrfs*15	LP	Proband	F	29	+
	c.560T>G, p.Leu187Arg	LP	Proband	F	44	+
	c.628C>T, p.Arg210*	P	Proband	M	42	+
	c.791+1G>A, p.(?)	P	Proband	F	46	+
			Brother	M	55	+
	c.850C>T, p.Arg284Cys	P	Proband	F	42	+
	c.1018C>T, p.Arg340*	P	Proband	F	31	+
	c.1199T>G, p.Met400Arg	LP	Proband	M	32	+
	c.1373dupG, p.His459Profs*210	P	Proband	M	69	+
			Father	M	n.a.	+
	c.1394C>A, p.Ala465Glu	LP	Proband	M	33	+
	c.1486delG, p.Ala496Profs*60	LP	Proband	M	71	+
	c.1602delA, p.Lys534Asnfs*22	P	Proband	M	41	+
	c.1779delA, p.Gln593Hisfs*7	LP	Proband	M	44	+
	c.1807delG, p.Val603*	P	Proband	F	44	+
	c.2089C>T, p.Arg697Trp	LP	Proband	F	39	+
	c.2278T>C, p.Cys760Arg	LP	Proband	M	28	+
	c.2284C>T, p.Gln762*	P	Proband	M	30	+
	c.2487dupA, p.Asp830Argfs*21	LP	Proband	M	43	+
Smith et al. (2015) [33]	c.570delT, p.Phe190Leufs10	P	Proband	M	37	+
			Proband	M	37	+
	c.2284C>T, p.Gln762*	P	Proband	F	20	+
			Proband	M	17	+
			Proband	F	25	+
	c.605T>G, p.Met202Arg	VUS	Proband	F	44	+
			Proband	M	23	+
	c.964G>T, p.Glu322*	LP	Proband	M	38	+
	c.1483dupG, p.Glu495Glyfs*174	LP	Proband	F	53	+
			Proband	F	25	+
	c.1175C>T, p.Ala392Val	VUS	Proband	F	20	+
	c.401-2A>G, p.(?)	P	Proband	n.a.	60	+
	c.509G>A, p.Arg170Gln	P	n.a.	n.a.	39	+
			n.a.	n.a.	66	+
	c.842delC, p.Pro281Argfs*70	P	Proband	n.a.	52	+
	c.856G>A, p.Gly286Arg	LP	Proband	n.a.	51	+
	c.1353+1G>A, p.(?)	LP	Proband	n.a.	15	+
	c.1583T>G, p.Leu528Arg	LP	Proband	n.a.	43	+
	c.1893delG, p.Lys632Serfs*20	P	Proband	n.a.	28	+
	c.1961A>G, p.Asp654Gly	LP	Proband	n.a.	45	+
	c.2002G>T, p.Asp668Tyr	LP	Proband	n.a.	22	+
	c.2062C>T, p.Arg688Cys	LP	Proband	n.a.	16	+
	c.27delG, p.Gln10Argfs*15	P	Proband	n.a.	48	+
	c.1785+2delT, p.(?)	LP	Proband	n.a.	43	+
Farschtschi et al. (2016) [38]	c.975delG, p.Gln325Hisfs*26	LP	Proband	M	30	+
Smith et al. (2017) [25]	c.1210 G>A, p.(Gly404Arg)	P	Proband	M	39	+
Louvrier et al. (2018) [27]	c.27delG, p.Gln10Argfs*15	P	Proband	F	n.a.	+
			Proband	n.a.	48	+
	c.264-13G>A, p.Lys89Cysfs*16	LP	Proband	M	n.a.	+
			Son	M	n.a.	+
	c.353G>A, p.Arg118His	LP	Proband	n.a.	n.a.	+
			Proband	n.a.	n.a.	+
			Proband	n.a.	n.a.	+
			Proband	n.a.	n.a.	+
	c.428delA, p.Asn143Ilefs*4	LP	Proband	n.a.	n.a.	+
	c.652-34G>T, p.(?)	VUS	Proband	n.a.	n.a.	+
	c.685_692delTGCAACTT, p.Cys229Profs*28	LP	Proband	n.a.	n.a.	+
	c.764T>G, p.Leu255Arg	VUS	Proband	n.a.	n.a.	+
	c.842C>T, p.Pro281Leu	LP	Proband	n.a.	n.a.	+
	c.891T>G, p.Tyr297*	LP	Proband	n.a.	n.a.	+
	c.967_980delGTCGTCCAGCCCAG, p.Val323Leufs*9	P	Proband	n.a.	n.a.	+
	c.993+1G>T, p.(?)	P	Proband	n.a.	n.a.	+
	c.1015G>T, p.Glu339*	LP	Proband	n.a.	n.a.	+
	c.1084C>T, p.Arg362*	P	Proband	n.a.	n.a.	+
	c.1126C>T, p.Gln376*	P	Proband	n.a.	n.a.	+
	c.1260+2T>C, p.(?)	LP	Proband	n.a.	n.a.	+
	c.1299C>G, p.Tyr433*	LP	Proband	n.a.	n.a.	+
	c.1394C>T, p.Ala465Val	VUS	Proband	n.a.	n.a.	+
	c.1396C>T, p.Arg466Trp	P	Proband	n.a.	n.a.	+
	c.1576C>T, p.Gln526*	P	Proband	n.a.	n.a.	+
	c.1957dupC, p.Gln653Profs*16	LP	Proband	n.a.	n.a.	+
	c.1964T>A, p.Met655Lys	VUS	Proband	n.a.	n.a.	+
	c.2063G>A, p.Arg688His	LP	Proband	n.a.	n.a.	+
	c.2407-2A>G, p.(?)	P	Proband	n.a.	n.a.	+
	c.2429G>A p.Arg810Gln	LP	Proband	n.a.	n.a.	+
	c.1-?_2523+?del	P	Proband	n.a.	n.a.	+
Kehrer-Sawatzki et al. (2018) [28]	c.2247C>A, p.Tyr749*	P	Proband	n.a.	n.a.	+
	c.1653C>G, p.Tyr551*	P	Proband	n.a.	n.a.	+
	c.975delG, p.Gln325Hisfs*26	LP	Proband	n.a.	n.a.	+
	c.321-2A>C, p.(?)	LP	Proband	n.a.	n.a.	+
	c.1018C>T, p.Arg340*	P	Proband	n.a.	n.a.	+
	c.628C>T, p.Arg210*	P	Proband	n.a.	n.a.	+
	c.1792T>C, p.Cys598Arg	VUS	Proband	n.a.	n.a.	+
Jordan et al. (2018) [29]	c.73delG, p.Ala25Profs*17	P	Proband	n.a.	n.a.	+
	c. 1084C>T, p.Arg362*	P	Proband	n.a.	n.a.	+
	c. 1396C>T, p.Arg466Trp	P	Proband	n.a.	n.a.	+
	c.2350_2360del, p.Gln784Hisfs*63	P	Proband	n.a.	n.a.	+
	c.1786–1G>A, p.(?)	LP	Proband	n.a.	n.a.	+
Deiller et al. (2019) [39]	c.1126C >T, p.(Gln367Ter)	P	Proband	F	43	+
	c.264-13G>A, p.Lys89Cysfs*16	LP	Proband	F	70	+
Alaidarous et al. (2019) [22]	c.692delT, p.Phe231Serfs*21	LP	Proband	F	23	+
	c.764 T > G, p.Leu255Arg	VUS	Proband	F	38	+
	c.264-13G > A, p.Lys89Cysfs*16	LP	Proband	F	28	+
Herrero San Martin and Alcala-Galiano (2020) [30]	c.1203C>G, p.Tyr401*	LP	Proband	M	54	+
Muthusamy et al. (2021) [31]	c.231delA p.Asp78Metfs*23	LP	Proband	M	19	+
Loh et al., 2022 [32]	c.1768C > T; p.Gln590*	P	Proband	F	48	+
	c.1210G > A; p.Gly404Arg	P	Proband	M	28	+
Current case 1	c.979delA, p.Ser327Alafs*24	LP	Proband	M	55	+

## Data Availability

All relevant data are available from the corresponding author upon request.

## Supplementary Materials

The following supporting information can be downloaded at: https://www.mdpi.com/article/10.3390/genes15070916/s1, Supplementary Table S1. Comparison between clinical evidences between Noonanan syndrome and the autosomal dominant LZTR1-Noonan syndrome.
